# How interaction molds semantics: The mood functions of Chinese “sum-up” adverbs

**DOI:** 10.3389/fpsyg.2022.1014858

**Published:** 2022-12-30

**Authors:** Jingyao Song

**Affiliations:** College of Foreign Languages, Shanghai Maritime University, Shanghai, China

**Keywords:** mood adverbs, pragmatics, TCSOL, corpus, modern Chinese linguistics

## Abstract

“Hezhe” (合着), “ganqing” (敢情), and “nao le bantian” (闹了半天) are common mood expressions in modern Chinese which have a common function of summarizing the information before (so called ‘sum-up’) as well as similar pragmatic functions. Study on these mood adverbs could reveal how the interactional mechanism molds the original semantic meanings of mood words and leads to new pragmatic functions. Five verbal corpora are applied to collect the real materials of usage containing the above three mood adverbs. Functional analysis and data statistics have been carried out to categorize the pragmatic functions of these words, calculate their distributions, and reconstruct their evolutional approach through an interactional perspective. We have found a core theoretical viewpoint that the similar functions of the three words emerged through the same pragmatic mechanism called “violation”. These functions are: (1) “unexpectation” from the violation of psychological expectation; (2) “criticism” from the violation of universal principles; (3) “humor” from the violation of communicative principles. A statistic work of several corpora showed that these functions of the above three words appear broadly in verbal materials, with differences in their proportions according to the communication types and genres. In Chinese teaching to speakers of other languages, more attention should be paid to these words stemming from dialects, especially during intermediate and advanced levels.

## Introduction

### What are “sum-up” adverbs?

As a highly isolated language, Chinese doesn't have mood inflections like subjunctive expressions in English and other Indo-European languages. However, emotion, attitude, intimacy, judgment, and so on of the speaker could be expressed by some words such as adverbs and some adverbial phrases, known as “mood adverbs.” However, in other situations, an adverb that originally holds a certain meaning could be attached with some “mood meaning,” thus transforming it into a mood adverb. Such transformation would happen habitually during daily communications before it was fixated and became the prevalent or even dictionary meaning of the word itself. In other words, such mood meanings have been molded during language interactions.

In the intermediate and advanced levels of Teaching Chinese to Speakers of Other Language, or TCSOL, sometimes real audio-visual materials are used as pedagogical materials, such as Chinese interview program clips. During class, students often raise questions about the meaning and usage of some words with mood meaning in the materials, usually colloquial rather than formal. These words do not appear in textbooks, and the TCSOL syllabus does not fully incorporate them. It is difficult for students to fully understand and even master their usage only by consulting dictionaries or other reference books. Especially, some colloquial words originating in dialects have not yet been included in dictionaries. Among them, several such mood adverbs with similar functions have attracted our attention, for example:

(1)     进我们自己的家,         还需要往你口袋里扔钱,   合着里外都让您赚了,    这合适吗？

          jin         women     ziji     de            jia,         hai

          enter     our           own    of            home     still

          xuyao   wang        ni       koudai     li

          need     to             your   pocket     inside

          reng       qian,        **hezhe**                     liwai                       

          throw     money     **totally speaking**    inside and outside  

          dou        rang         nin

          all          let            you

          zhuan     le,                                           zhe     

          earn       (perfective aspect particle)     this     

          heshi      ma?

          proper   (question article)

          *To enter our own house, we need to throw money into your pocket, so totally speaking, you earn money either way. Is this proper?* (from a news program on CCTV).

(2)     一千五百块钱, 我还当是稿费, 后来我才看明白, 敢情上杂志上发表您的论文, 是您要给杂志钱。

          yiqianwubai       kuai                       qian,         wo

          1500                  (currency unit)       money      I

          hai                     dang                       shi            gaofei,

          originally          think                       be             royalties

          houlai               wo        cai             kan       mingbai,

          later                  I           just             see       clearly

          **ganqing**           shang   zazhi          shang

          **you dare say**   go         magazine   on

          fabiao     nin             de       lunwen,   shi   nin   yao

          publish   you            of        paper       be   you   need

          gei         zazhi          qian.

          give       magazine   money

          *I had thought this 1500 yuan was the royalties (given to the author). Only later did I understood, you dare say, that if you'd like to publish your paper on the magazine, it is you who should pay money* (from a talk show on Phoenix Satellite TV).

(3)     闹了半天, 是这么回事儿, 既然这吉祥号这么惹麻烦, 您就不能换一个吗？

          **Nao le bantian,**        shi     zheme

          **after so much fuss**   be      so

          hui                             shier,

          (measure word)         issue

          jiran    zhe   jixiang         hao         zheme   re        mafan,

          since   this   auspicious   number   so         incur   trouble

          nin    jiu                        bu                          neng   huan

          you   just                       not                         can     change

          yi      ge                         ma?

          one   (measure word)   (question article)

          *After so much fuss, we finally make out the truth. Since the auspicious phone number incurs so much trouble, why can't you change for another one?* (from a news program on Beijing TV).

From the examples above, we can see words and phrases like “hezhe” （合着）, “ganqing” （敢情）, and “nao le bantian” （闹了半天）, and they express a basic meaning similar to “conclude” in colloquial Chinese to summarize the information that appeared before their clause. Yet, they are different from the typical “conclude” adverbs and phrases such as “zongzhi (总之)” (in a word) and “zong de lai shuo (总的来说)” (generally speaking), in that they don't acquire such meaning through the morpheme or elements that they are composed of such as “总” (total) and in that they contain certain kinds of mood besides the basic semantic meaning, e.g., beyond expectation, dissatisfaction, criticism, etc., which the typical “conclude” words don't have. Therefore, these words should form an independent category as they perform similar pragmatic functions during interactions above their basic semantic meaning, that is, these words can be regarded as **mood** adverbs in a broad sense, and at the same time, they all complete the function of summarizing the situation. We refer to these words as “sum-up” mood adverbs.^1^

Mood elements in modern Chinese are a kind of collection with different origins, complex internal compositions, and diverse meanings and functions. There are different views on the classification of the Chinese mood system (Lv, 1982; Wang, 1985; Gao, 1986; He, 1992; Qi, 2002) and the classification of mood words (Zhang, 2000; Qi, 2003). In particular, the classification and functional generalization of mood words have been discussed for a long time, but a consensus has not yet been reached. The reason for this complex state is that the “mood” gradually obtains its pragmatic meaning and function in the process of real communication.

Social interaction and communication are the original natural habitat of language. We should understand the structure and application of language from the actual verbal communication (Fang, 2005; Couper-Kuhlen and Selting, 2018; Fang et al., 2018; Zeng, 2021). For every specific language component, their research should not be separated from the conversation environment in which they are located, and we should study “dynamic” language (Zhu, 2021). For various mood adverbs mainly produced and used in spoken language, their synchronic performance and functions also “emerge” in the communication within a conversation from the diachronic perspective, thus gradually forming and shaping the language system. Although many mood words stemmed from different sources, they gradually form pragmatic conventions through their use in communication, and according to their pragmatic functions, similar mood words can be grouped together. Therefore, it is necessary to study the specific functional categories to which each specific mood word belongs, explain the different “emerging” paths of similar pragmatic functions in the same category, explore the aggregation mechanism of each category, and then piece together the classification panorama of mood words themselves.

To train learners' oral Chinese communication ability in a real context, it is also necessary to make an in-depth study of these colloquial mood adverbs and analyze their semantic and pragmatic functions by using real corpora. This study, therefore, undertook a pragmatic study on the mood adverbs with “sum-up” meaning such as “hezhe,” “ganqing,” and “nao le bantian” and explained the emergence and development of the pragmatic functions of these words in communication, to provide a reference for international Chinese education.

### Research review

This research selected a special category of mood adverbs, whose core meaning can be summarized as “sum-up.” Representative words include “hezhe,” “ganqing,” and “nao le bantian (nao le guiqi).” These words originated from different sources, but they “emerge” with similar pragmatic functions in communication, and their functions have not been accurately recognized or described so far.

Studies on this kind of mood words are few, most of which are dissertations, and their conclusions are quite similar. Qi (2008) defined “hezhe” as a colloquial word in Beijing dialect and summarized its five meanings, of which the last one is “that the speaker makes corresponding reasoning or assumptions about things on the premise of integrating the existing conditions, or knows something according to the summary,” and believes that this usage is equal to “yuanlai” (原来, turn out to be). Qi's study only listed the different usages of “hezhe” at the semantic level, without considering the pragmatic mechanism. The master's thesis of Liu (2012) followed the classification of Chinese adverbs from Zhang (2000), classified “hezhe” as a commentary adverb, and summarized its semantic function into two aspects: in terms of message transmission, its function is “interpretation of known real news by tracing its origin,” and in terms of modality, its function is “emphasis” and “in-depth probe.” The pragmatic functions of “hezhe” are the evaluation function, emphasis function, and textual function. Fang (2018) summarized the pragmatic functions of “hezhe” into three categories: “objective description” “subjective cognition,” and “negative evaluation” in his doctoral thesis, and believed that its expression of negative evaluation is a prescriptive meaning that has “emerged” in interaction.

There are also several specialized research studies on “ganqing.” The master's thesis of Wang (2014) also followed the theoretical framework of Zhang (2000) and summarizes the function of “ganqing” into two aspects: semantic function and pragmatic function. The semantic function includes communication functions such as explanation, assertion, speculation, and summary, as well as mood functions such as emphasis and in-depth probe. The pragmatic function includes evaluation, indicating presupposition, expression, emphasis, and textual function. Han (2014) summarized the meaning of “ganqing” into three kinds: (1) “tracing the origin thus making explanation,” (2) assertions that reinforce affirmation, and (3) indicating a speculative question. The motivation and mechanism of grammaticalization of “ganqing” mainly include frequency principles, analogy principles, rhythm principles, and pragmatic factors. Wu's master's thesis (Wu, 2016) compared “ganqing” with “yuanlai,” and “biding (必定)” (for sure) and “nandao (难道)” (is it really that…?), and held that they have something in common in semantics, but “ganqing” expresses stronger feelings and stronger subjectivity. This research also summarized the main error types of “ganqing” in Chinese as second language acquisition. Nan (2019) summarized the evolving process of “ganqing” from a diachronic perspective and believed that the development of “gan” (dare) and “qing” (emotion) combined from independent words to verb-object phrases, and then evolving into commentary adverbs is the result of the joint influence of the meaning of the words themselves and the external communication situation.

As to other such mood adverbs, no specialized research has been seen. The comparative research of two or more such mood adverbs is also limited to “hezhe” and “ganqing.” Among them, Fang (2018) believes that the two words can be interchanged when expressing the positive judgment function, while when “hezhe” is used as an attitude marker and “ganqing” is used to express the interrogative judgment function, they cannot be replaced with each other. Yang's master's thesis (Yang, 2019) conducted a specialized study on these two words and found that they overlap in semantics, but “ganqing” has richer semantic connotations and more flexible syntactic distribution.

Generally speaking, the research on such mood adverbs focuses mainly on the two words “hezhe” and “ganqing.” The conclusions show the following features: (1) Stepping from the parallel summary of multiple meaning items to the distinction between semantic and pragmatic levels; (2) Looking for the motivation of word evolution from the inside and outside aspects of words, and taking context, communication, and interaction into account; (3) Actively exploring other theoretical perspectives outside the framework of traditional Chinese grammar research.

Besides the small scale, existing studies are still wanting in several aspects: First, the scope of words studied is relatively narrow, and still fewer comparisons among different mood adverbs. Second, the mechanism of pragmatic function is not fully explored, thus lacking a thorough explanation of the motivation behind the gradual occurrence of the various pragmatic functions. Third, is the lack of corpus work. The existing research works using corpora to investigate the meaning and usage of such mood adverbs are limited to dissertations, and most of them wield written corpora. The analysis and statistics from these studies are not comprehensive enough to fully reflect the distribution and use of these kinds of mood adverbs.

### Research method

This paper studies the “sum-up” mood adverbs, selecting “hezhe (合着),” “ganqing (敢情),” and “nao le bantian (nao le guiqi) (闹了半天/闹了归齐)” as examples, uses the oral corpora as the research material, explains the pragmatic function and “emergent” principle of each word through the analysis and statistics from oral conversations, and locates the common mechanism behind the clustering of such mood adverbs. We further try to order the semantic and pragmatic conditions of each word and make statistics and induction on the distribution of different usages, so as to provide a reference for TCSOL.

The corpora used in this paper include five parts: (1) the “Media Language Corpus” (MLC) of Communication, University of China, which contains 34,039 transcribed texts of radio and television programs from the year 2008 to 2013, with a total number of 241,316,530 characters. (2) The TV script of <I Love My Family>, the dialogue of which is Beijing dialect in the 1990s, with a total number of 606,950 characters. (3) CCL Corpus of Peking University, “Oral,” “Artistic TV and movie script,” “Crosstalk and short sketch” part of “Modern period,” and “Script” part of “Contemporary period.” (4) General Balanced Corpus of Modern Chinese of China's National Language Commission. (5) “Dialogue” part from BCC Corpus of Beijing Language and Culture University. Because the above corpora are real or imitations of daily conversations, this study can reflect the use of mood adverbs in real communication. For reference, during the research process, we still deployed the “Ancient Chinese” part of the CCL corpus and the HSK corpus from Beijing Language and Culture University as additional resources of ancient Chinese languages as well as of learners of Chinese as a second language.

The workflow of this study is as follows: (1) search each target word in each corpus, manually filter the cases where it is used one by one and remove the cases that are not the object of this study, such as “他合着眼躺在床上” (“he lies on the bed with his eyes closed” in which “hezhe” means “closing”), leaving only valid cases. (2) Invite two other linguists whose mother tongue is Chinese to select ten cases from each of the three groups of valid cases to discuss their pragmatic functions and unify the criteria for judgment. (3) The author and two judges, respectively, judge the pragmatic functions of all cases. (4) By comparing the three judgments, each pragmatic function can be determined only after at least two people agree. If there are three different results, the case should be discussed further and then determined (this did not happen in the actual process).

## Analysis of pragmatic functions of “sum-up” mood adverbs

Searching through the corpora, 1,284 valid cases were found, including 368 cases of “hezhe,” 833 cases of “ganqing,” and 83 cases of “nao le bantian” (including 1 case of “nao le guiqi“). The following is a semantic and pragmatic analysis of these words combined with examples from the corpora.

### Semantic and pragmatic analysis of “hezhe” (合着)

The Modern Chinese Dictionary (7^th^ Edition) (Dictionary Compilation Office, 2016) does not include “hezhe” as a word, which may be because the origin of “hezhe” has a more dialect feature. Based on the basic meaning of “he” (合): amount to, or add up to, combing the previous research and analyzing the frequency distribution of using cases from corpora, we believe that the basic semantic meaning of “hezhe” is “a summary of the above information,” especially the conclusion of digital calculation. This can be regarded as the basic usage of “hezhe” and also the origin of its “sum-up” function. In ancient vernacular from CCL corpus from Ming Dynasty to the year 1949, we found 67 cases of “hezhe” yet all belonged to the basic meaning or served as a simple verbal phrase. Therefore, we believe that this basic meaning was the original point of the other pragmatic functions. In contemporary corpora, this basic meaning is still used in the following examples:

(4)     年薪4800块, 合着一个月才400元。

          Nianxin            4800     kuai,                   **hezhe**

          annual salary                (currency unit)    amount to

          yi      ge                        yue          cai

          one   (measure word)   month     only   

          400 yuan.

          (currency unit, equal to ”kuai“)

          *The annual salary of 4800 yuan amounts to 400 yuan per month*.

(5)     5个小时, 1200条短信, 这合着4秒钟就发一条短信啊。

          5 ge                     xiaoshi,   1200 tiao             

          (measure word)   hour        (measure word)   

          duanxin,            zhe   hezhe

          short message   this   amount to

          4 miaozhong               jiu           fa               yi       

          second                        only         send           one   

          tiao                             duanxin             

           (measure word)          short message   

          a.

          (exclamation mark)

          *5 hours, 1200 short messages sent, this amounts to 4 seconds per message*.

The above cases use the basic meaning of “hezhe,” in which (4) represents the calculation result of money, (5) represents the calculation result of time, and (6) represents the calculation result of proportion. It should be noted that when “hezhe” is used to represent the calculation result, it is not necessarily the total result obtained by adding, but can be extended to other calculation methods as well. For example, (4) and (5) are the result of division.

In addition to expressing the calculation results, the most common usage of “hezhe” is to convey different pragmatic meanings, including:

(6)     王先生首付款才交了18万, 合着首付一成多。

          Wang                 xiansheng   shoufu kuan         cai

          (family name)   Mr.              down payments    only

          jiao                    le

          pay                    (past tense)

          18 wan,

          ten thousand

          hezhe          shoufu                 yi      cheng   duo.

          amount to   down payments   one   10%      more

          *Mr. Wang just paid 180,000 yuan for down payments, which is only a 10% of the total price*.

(7)     我选择这两位大爷大娘, 合着他们四个都过关啦。

          Wo        xuanze   zhe     liang    wei

          I            choose   these   two     (measure word)

          daye      daniang,

          uncle     aunt

          hezhe

          unexpectedly

          tamen     si          ge                       dou   guoguan

          they        four     (measure word)   all     pass

          le

          (past tense and exclamation)

          *I will choose the uncle and aunt, and unexpectedly they four have all passed the test*.

(8)     合着刚刚放进存款机的16500块, 压根就没存进去。

          Hezhe               ganggang   fang                 jin

          Unexpectedly   just now     put                   in    

          cunkuanji         de               16500 kuai,

          ATM                of                (currency unit)

          yagen          jiu      mei    cun         jinqu.

          absolutely   just    not     deposit   into

          *Unexpectedly, the 16500 yuan put into the ATM just now has totally not entered the account*.

(9)     合着里外都让您赚了, 这合适吗？

          Hezhe    liwai                        dou   rang   

          Totally   inside and outside   all     let

          nin         zhuan                       le.

          you        earn                          (past tense)

          zhe     heshi             ma?

          This   appropriate   (question particle)

          *Totally speaking, in both situation you made the profit. Is it appropriate?*

(10)   合着您养他就是为了吃他, 他是猪还是鸡啊？

          Hezhe                   nin   yang   ta    jiushi   

          totally speaking   you   raise   he   is          

          weile                    chi    ta,

          in order to            eat    he

          ta    shi   zhu   haishi   ji

          he   is     pig    or        chicken

          a?

          (question particle)

          *Totally speaking, you brought him up just to depend on him one day. Is he a pig or a chicken?*

(11)   这边写着, 这边手插电板里, 都是这样写稿子, 合着成电动的了。

          Zhe    bian     xie       zhe,                          zhe

          this    side     write   (progressive tense)   this   

          bian   shou    cha     dianban li,

          side    hand   stick   socket inside

          dou    shi   zheyang    xie       gaozi,

          all      are   so             write    article

          hezhe                     cheng           diandong

          totally speaking     become        electrified

          de                           le.

          (auxiliary word)    (past tense)

          *We will write while the other hand is stuck into the socket, and that's how we write articles. Totally speaking, this has become an electrified way of writing*.

(12)   后来我说合着这花是给你买的我说, 她说来个玫瑰浴自己。

          Houlai   wo   shuo   hezhe                   zhe   

          After      I      say     totally speaking   this   

          hua        shi   gei      ni      

          flower   is     give    you   

          mai       de

          buy       (auxiliary word)

          wo                       shuo,      ta      shuo       lai        

          I                           say         she     say         make   

          ge                        meigui   yu      gei ziji.

          (measure word)   rose       bath   give self.

          *Later I said, totally speaking, you bought the flower for yourself. She said she would use the flowers to have a rose bath for herself*.

Among the above examples, in (7) and (8) the pragmatic function of “hezhe” is to convey the mood of “unexpected,” and the reason behind “unexpected” is that the information violates the speaker's psychological expectation. It should be noticed that here we use “expectation” not as “hope” or “wish,” but only when the expression following “hezhe” is against the original guess or imagination of the speaker shall we mark it as “unexpected,” which is different from the situation that the speaker himself/herself have hoped or wished to happen, or what is beneficial to him/her. In detail, “hezhe” in (7) means that “I” didn't expect old couples could pass the test, and “hezhe” in (8) means that the money I thought I had saved was unexpectedly not deposited. The “hezhe” of (9) and (10) complete a pragmatic function of “criticism,” which is caused by violating the generally recognized common sense. For example, “hezhe” in (9) criticizes that people listening to it should not make money on both sides, and “hezhe” in (10) criticizes that people listening should not regard their son as a source of money. The “hezhe” of (11) and (12) performs the pragmatic function of producing the effect of “humor,” which is based on the intentional violation of communication conventions. In (11), the humorous effect comes from the intentional violation of the common sense shared by both parties (it is impossible to write in this “electric” way), and the humor of (12) comes from the intentional violation of the facts shared by both parties (the flowers are actually bought by her as a present for ”me“).

It can be seen that when “hezhe” is used as a pragmatic function, its core mechanism lies in violating people's particular habitual thinking or certain laws. How did this pragmatic function develop? We believe that this can be traced back to the role of verbal communication. Because the basic meaning of “hezhe” is used to express the results achieved by calculation, and in daily verbal communication, the results of “unconventional” or “beyond convention” will attract people's attention and have a higher value of information exchange. Therefore, “hezhe” has gradually emerged with a series of pragmatic functions based on “violation” in communication, which has also transformed it from a simple quantitative adverb to a mood adverb. This is a typical example of the effect of verbal communication on the development of the pragmatic function of adverbs.

**Figure 1:**
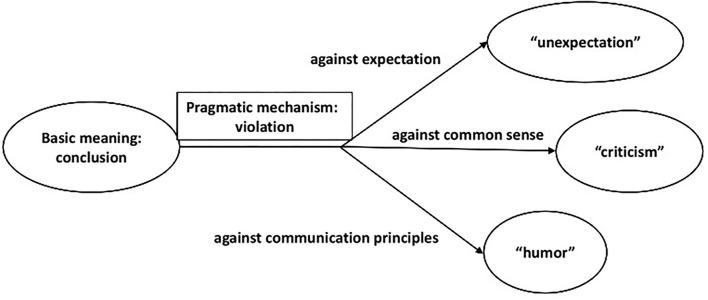
The meanings and pragmatic functions of “hezhe.”

The above meanings and pragmatic functions of “hezhe” are shown in Figure 1.

**Figure 2:**
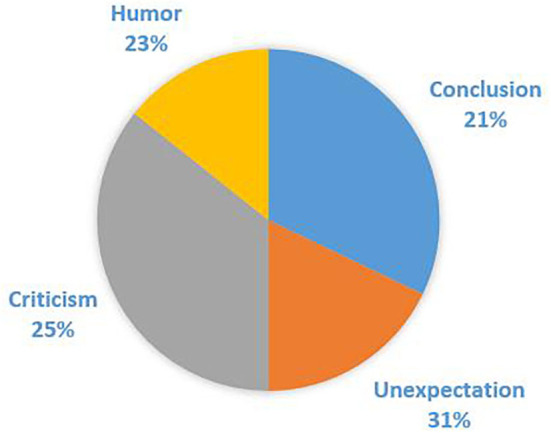
Distribution of “hezhe.”

Among the 368 cases of “hezhe” in corpora, 77 cases were used as the basic meaning of “conclusion,” while 291 cases were used as pragmatic functions, including 116 cases expressing “unexpectation,” 91 cases of “criticism,” and 84 cases of “humor.” Their distribution is shown in Figure 2.

It can be seen that the basic meaning “conclusion” of “hezhe” is still used, yet its proportion ranks lowest compared to other mood functions. Among other usages generated by communication, “unexpectation” is the most frequent pragmatic function, and the proportions of “criticism” and “humor” are a little lower.

In addition to the adverbs of “conclusion,” are there other words that can repeat this path and develop similar pragmatic functions in verbal communication? In the following sections, we will explore the other adverbs with similar functions so as to make sure whether they had the same evolving process as “hezhe.”

### Semantic and pragmatic analysis of “ganqing” (敢情)

The basic semantic meaning of “ganqing” in the Modern Chinese Dictionary (7th Edition) is “indicating the information that has not been found before.” According to previous research and case distribution from the corpora, we summarize the basic meaning as “discovery,” and such discovery always happens on the basis of the information before, thus rendering it the basic function of “sum-up.” In the ancient Chinese corpus, nearly two-thirds of the “ganqing” cases belong to this basic meaning. Of the 833 cases in the contemporary corpora, there are 422 cases of this basic usage. For example:

(13)   记者仔细一问, 敢情是家里孩子出状况了！

          Jizhe           zixi               yi       wen,      ganqing

          reporter      thoroughly    once   ask         the truth is

          jia               li

          home          inside

          haizi           chu           zhuangkuang   le!

          children      happen     issue              (past tense)

          *The reporter inquired them thoroughly, and found out the truth that their children had some emergent issues at home*.

(14)   听了半天, 敢情人家这楼上一直有个心结。

          Ting      le                   bantian,            ganqin

          listen     (past tense)    half a day        the truth is

          renjia     zhe

          they       this

          loushang      yizhi      you      ge

          upstairs        always   have    (measure word)

          xinjie.

          obsession

          *Listening for a long time, we finally found out the truth: the people upstairs have always had something unsatisfied*.

(15)   齐先生一查, 原来是当地有人跟他同名同姓, 敢情是银行弄错了。

          Qi                      xiansheng                     yi

          (family name)    Mr.                               once

          cha,                    yuanlai                         shi

          investigate          turn out to be    is

          dangdi

          local

          you                     ren                               gen

          have                   person                          with

          ta                        tongmingtongxing,      ganqing

          him                    have the same name     the truth

          shi

          is

          yinhang         nong        cuo         le.

          bank              do            wrong     (past tense)

          *Mr. Qi had made some investigation and it turn out to be that there's a local person with the same name as him, so the truth is that the bank had made a mistake*.

The above three cases all indicate that some information was found after certain inquiries or investigations. In actual verbal communication, the information often found is the result that is inconsistent with some expectations held by the speaker before. Therefore, some functions similar to “hezhe” appears in pragmatics, which can be divided into several categories depending on the object it violates. For example:

(16)   专家的解释我们倒是第一次听说, 以前光害怕手机辐射人了, 敢情人也能干扰手机啊。

          Zhuanjia               de                      jieshi                 women

          Expert                   of                      explanation       we

          daoshi                   diyici                tingshuo

          are                         the first time     hear

          yiqian                    guang                haipa                 shouji

          before                    only                  fear                    cellphone

          fushe ren le

          radiation                people               past tense)

          ganqing                  ren                    ye                       neng

          unexpectedly         people               also                    can

          ganrao                    shouji

          interfere                 cellphone

          a.

          (exclamation particle)

          *The explanation of experts is quite new to us, since we only fear the radiation of cellphones to people before. Unexpectedly, people can also interfere cellphones*.

(17)   原来光以为执著是个好词儿, 敢情执着如果用错了地方, 也会给人惹麻烦。

          Yuanlai               guang                    yiwei           zhizhuo

          before                 only                      think             persistent

          shi                      ge                          hao               ci’er

          is                        (measure word)     good             word

          ganqing                       zhizhuo           ruguo              yong

          unexpectedly      persistent                 if                     use

          cuo                      le                             difang,

          wrong                 (past tense)              place

          ye        hui      gei       ren                 tian      mafan.

          also     will     give     peole              add      trouble

          *I only thought “persistent” is a good word before. But unexpectedly, if persistence is used in wrong place, it could make people trouble, too*.

(18)   可让他没有想到的是, 敢情这足球还真不是一般人能搞的, 扔钱都不带响。

          Ke               rang                        ta          meiyou

          but               let                           him       not

          xiangdao     de                           shi,

          expect         (auxiliary word)     is

          ganqing              zhe        zuqiu                     hai

          unexpectedly      this       soccer                    still

          zhen                    bushi    yiban                     ren

          really                  isn't      ordinary                 people

          neng                    gao      de,

          can                      do         (auxiliary word)

          reng       qian         dou     bu       dai        xiang.

          throw     money     all       not      with     sound.

          *But something surprised him. Unexpectedly, the soccer is not a sports for ordinary people, to which your invest disappeared without any trace*.

In examples (16–18), the new information found by the speakers is inconsistent with their original cognition, in (16) the speaker thought that “only mobile phones can radiate people;” in (17) the speaker thought that “persistence is a good thing;” and in (18) “he” thought that “ordinary people can invest in football.” Therefore, the pragmatic function of “ganqing” is to highlight the “violation” of these previous expectations. This pragmatic function can be summarized as “unexpectation,” which is similar to the first type of function developed by “hezhe” in communication. There are 109 cases of these functions in the corpora.

The second kind of pragmatic function of “hezhe,” which indicates the violation of the recognized knowledge of things or actions, resulting in the meaning of “criticism,” also appears in the corpora. For example:

(19)   毕竟他是经济学博士, 行贿的巴能军也是经济学博士, 敢情人家学经济主要脑子用这儿了。

          Bijing              ta               shi                             jingjixue

          after all            he              is                               economics

          boshi,               xinghui     de                              Ba Nengjun

          doctor              bribery      (auxiliary word)        (name)

          ye                     shi             jingjixue                   boshi,

          also                  is               economics                doctor

          ganqing            renjia        xue                            jingji

          you dare say     they          study                         economics

          zhuyao       naozi     yong   zher    le.

          mainly       mind      use      here    (past tense)

          *After all he is a Doctor of Economics, so is Ba Nengjun who has committed bribery. You dare say, they study economics just to use their knowledge here*.

(20)   这话翻译过来就是:邮政法只管邮政局, 不管快递公司。乖乖,

          敢情邮政法是国家邮政企业的专用马甲。

          Zhe                 hua         fanyi           guolai       jiushi:

          this                 words      translate     around      is

          youzhengfa    zhi           guan

          Postal Law     only         regulate

          youzhengju,     bu                      guan

          post office        not                     regulate

          kuaidi               gongsi.              Guaiguai,

          express             company           (exclamation particle)

          ganqing            youzhengfa       shi

          you dare say     Postal Law        is

          guojia               youzheng          qiye

          country             post industry     company

          de                           zhuanyong     majia

          (auxiliary word)     specialized     tag

          *These words mean: Postal Law regulates only the post offices, not express companies. Good heavens! You dare say, Postal Law is the special tool of national post companies*.

(21)   教育厅前些日子刚发文说义务教育阶段‘零收费’, 敢情不收学费改卖衣服了。

          Jiaoyuting                                           qian

          Department of Education                   before

          xie                                                       rizi

          (measure word of plural form)           day

          gang            fa                                     wen

          just              publish                             decree

          shuo            yiwujiaoyu                       jieduan

          say              compulsory education     stage

          “ling            shoufei,”                           ganqing

          zero             charge                               you dare say

          bu                shou                                  xuefei

          no                charge                              tuition fee

          gai               mai                                   yifu

          change        sell                                   clothes

          le.

          (past tense)

          *The decree issued by Department of Education days before said that no fees should be charged during the stage of compulsory education. You dare say, they change from charging tuition fees to selling clothes*.

In (19), the practice of “Doctor of Economics” violated the principle of “people who study economics should also abide by laws.” In (20), the post office's response violated the principle that “the Postal Law should apply to all express companies.” In (21), the practice of collecting clothing fees from students violated the provision of “zero charge in the compulsory education stage.” Therefore, “ganqing” in the above cases conveys the pragmatic function of “criticism” of such behaviors. There are 147 such cases in the corpora.

In the cases of “ganqing,” there are also practices of deliberately violating the conversational principles to obtain the effect of “humor.” For example:

(22)   爱尔兰政府首先回过味来了, 敢情对付金融危机就像鲁提辖三拳打死镇关西。

          Aierlan             zhengfu             shouxian

          Ireland              government       first

          huiguowei        lai                      le,

          understand        around               (past tense)

          ganqing             duifu                  jinrong

          you dare say     combat              financial

          weiji                 jiu                      xiang

          crisis                 just                    like

          Lu                        Tixia

          (family name)      (government position)

          san                       quan

          three                     fist

          da                         si

          beat                      die

          Zhen Guanxi.

          (nick name)

          *The Irish Government first understood that to combat financial crisis is just like Lu Tixia beating Zhen Guanxi to death with three hits (in Chinese traditional legend)*.

(23)   看看,一个个,全是不同年代的老式收音机,敢情就这么个组合音响啊？

          Kankan,                                        yi

          look                                              one

          gege                                             quan

          (measure word in plural form)    all

          shi                                                butong

          are                                                different

          niandai

          period

          de                          laoshi

          (auxiliary word)    old style

          shouyinji,              ganqing

          radio                      you dare say

          jiu                          zheme

          just                        such

          ge

          (measure word)

          zuhe yinxiang        a?

          stereo system        (exclamation particle)

          *Look. These, one by one, are all old style radios of different periods. So that's what you call a stereo system?*

(24)   鹅的确也能看家, 那敢情公鸡中的战斗机, 战斗的本领是从鹅那儿学会的。

          E            dique        ye                     neng        kan

          goose     surely       also                   can          protect

          jia,         na             ganqing

          home     then          you dare say

          gongji       zhong                         de                       zhandouji,

          rooster      inside                         (auxiliary word) fighter

          zhandou    de

          fight          (auxiliary word)

          benling   shi            cong                          e          naer

          ability     is              from                          goose  there

          xue         hui            de.

          learn       master      (auxiliary word)

          *The geese surely can protect your home as well. Thus, did the most brave fighters of the roosters learn their skills from the geese?*

Case (22) deliberately violated the Maxim of Quality from the four Cooperative Principles (Grice, 1975) and used a Chinese traditional story to describe the psychological activities of a foreign government, which achieved the effect of humor. Similarly, case (23) violated the fact that “several radios do not constitute a stereo system.” Case (24) violated the fact that a goose can't teach a rooster to protect the house. In these examples, “Ganqing” conveys the pragmatic function of “humor” in a way that violates the Maxim of Quality, that is, not to say what you believe to be false, or to say that for which you lack adequate evidence. There are 155 cases of this usage in the corpora.

**Figure 3:**
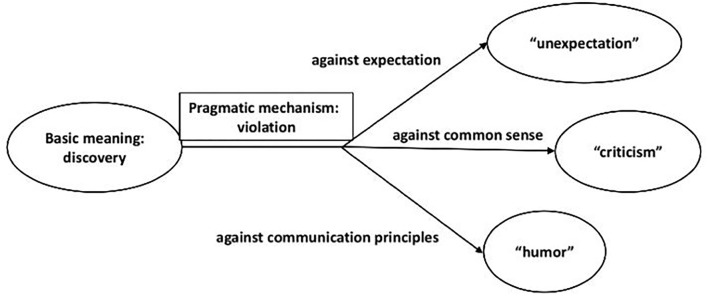
The meanings and pragmatic functions of “ganqing.”

To sum up, the adverb “Ganqing” started with the basic meaning “discovery,” through the “emergence” of pragmatic functions in communication similar to “hezhe,” and finally acquired almost the same pragmatic function system, including “unexpected” “criticism” and “humor,” as is shown in Figure 3.

**Figure 4:**
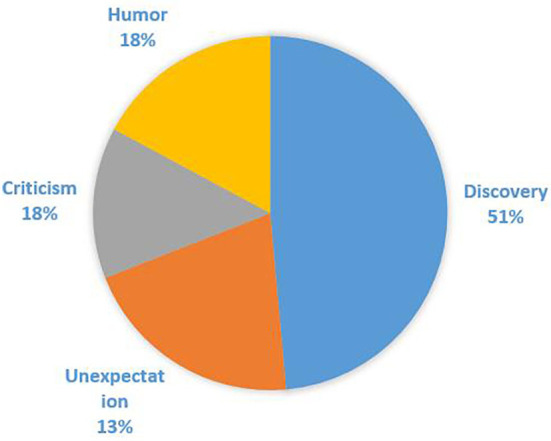
Distribution of “ganqing.”

Such usages of “ganqing” are distributed as shown in Figure 4.

Compared with “hezhe,” the original meaning “discovery” of “ganqing” occupies an absolutely dominant position, while the frequencies of the other three pragmatic functions decrease gradually from “humor” (155 cases), “criticism” (147) to “unexepectation” (109), but there is little difference.

It is the mechanism of verbal communication that enables “hezhe” and “ganqing” with different original meanings to obtain similar pragmatic functions. In real communication, the conclusion of facts and the discovery of new facts from the given information are easy to lead to the function of “sum-up,” then to the violation of some psychological expectations, common sense, and communication conventions. Therefore, the above two words have formed similar pragmatic functions. Whether this inference is true or not, we need to introduce other words to further verify.

### Semantic and pragmatic analysis of “nao le bantian(guiqi)” (闹了半天（归齐）)

”Nao le bantian” is also a common colloquial phrase. Its original meaning is “make trouble for a period of time (and get some results)“. In the ancient Chinese corpus, this original usage is seen in almost 70% of all instances. In the contemporary corpora, there are still examples of such usage:

(25)   咱们吵了半天闹了半天, 最后大家停下来都去捧读曹雪芹的原著, 我的目的就达到了

          Zanmen          chao            le                   bantian

          We                  argue          (past tense)    half a day

          nao                  le                bantian,

          make trouble  (past tense)  half a day

          zuihou    dajia          ting    xialai    dou   qu

          at last     everyone    stop   down    all     go

          peng     du        Cao Xueqin    de

          hold      read     (name)           (auxiliary word)

          yuanzhu

          original work

          wo              de                         mudi          jiu

          we              (auxiliary word)   purpose     just

          dadao          le.

          achieve       (past tense)

          *We have argued for a long time, and if at last everyone could stop and take an original work of Cao Xueqin to read, then my purpose will be achieved*.

(26)   因为现在闹了半天, 之所以闹了才派了几十个人去, 做了很大的文章。

          Yinwei     xianzai             nao                    le

          Because   now                  make trouble    (past tense)

          bantian,     zhisuoyi          nao

          half a day  the reason of   make trouble

          le                    cai                      pai        le

          (past tense)    only                    send     (past tense)

          ji                    ge                        ren       qu,

          several           (measure word)  people  go

          zuo                      le                   hen       da

          make                   (past tense)     very     big

          de                        wenzhang.

          (auxiliary word)  article

          *Not until the trouble had been made for a long time did several dozen people were sent and made it a big issue*.

(27)   小区的业主委员会就说这个管理公司什么什么有问题, 闹了半天, 闹到最后全部业主同意我们不交管理费。

          Xiaoqu             de                         yezhu

          housing estate  (auxiliary word)   owner

          weiyuanhui       jiu                        shuo

          committee         just                      say

          zhege

          this

          guanli                gongsi                 shenmeshenme

          manage             company              something

          you                    wenti,                  nao

          have                  problem               make trouble

          le

          (past tense)

          bantian,      nao                  dao        zuihou

          half a day   make trouble   to           last

          quanbu       yezhu              tongyi    women

          all               owner              agree     we

          bu       jiao          guanli     fei.

          not      hand in    manage  fee

          *The house owners of this estate said the management company had all kinds of problems. After making trouble for a long time, at last, all the owners agreed that they wouldn't hand in the management fee*.

In these cases, “nao le bantian” is a verb phrase in its original meaning, which has not been integrated into a special mood component. Therefore, it was not included in the scope of effective cases in our analysis and statistics. Except for these, there were still 82 cases of “nao le bantian” used as a mood adverb. In addition, we also found 1 case of “nao le guiqi,” a mood adverb with a similar meaning yet often appearing in the North dialect.

In these cases, the extended meaning closest to the original is the meaning of “conclusion” directly evolving from the meaning of “result,” that is, the meaning of “conclusion obtained over a period of time” was derived from the “result of trouble over a period of time.” This meaning is similar to the basic meaning of “hezhe.” There are eight such cases in the corpora. For example:

(28)   老板说得兴起, 可是, 闹了半天, 这价钱怎么样啊, 说着, 老板拿给我们一张价目表。

          Laoban          shuo                      de                         xingqi,

          boss               say                       (auxiliary word)   excited

          keshi,             nao la bantian,     zhe                       jiaqian

          but                 to sum up             this                       price

          zenmeyang

          how

          a,                            shuo     zhe,

          (question particle)  say      (progressive tense)

          laoban                     na        gei

          boss                        take      give

          women

          we

          yi      zhang                    jiamiu  biao.

          one   (measure word)     price    list

          *The boss talked excitedly. However, to sum up, how is the price? While talking, the boss gave us a price list*.

(29)   闹了半天, 你拐了半天, 怎么, 结论拐到哪去了？

          Nao le bantian,     ni              guai              le

          to sum up              you           go around     (past tense)

          bantian,                 zenme,     jielun

          half a day              how         conclusion

          guai              dao      na           qu     le?

          go around     to        where     go     (past tense)

          *To sum up, your talking has been winding for a long time, then, what is your conclusion?*

(30)   所以说这事要解决的话, 闹了归齐, 说到底, 还是个公德问题。

          Suoyishuo     zhe          shi                  yao

          So                  this          issue               will

          jiejue             dehua,     nao le guiqi,

          solve              if             to sum up

          shuo dao        di,

          speak to         essence

          hai              shi      ge                         gongde

          still             is        (measure word)   social morality

          wenti.

          problem

          *So if this issue wants to be solved, to sum up, after all, this is a problem of social morality*.

In case (28), the speaker hopes to conclude “what's the price” after a period of discussion; in (29), the speaker directly expresses his desire to “draw a conclusion“; in (30) “nao le guiqi” directly comes to the conclusion that “this is a problem of social morality.” In another direction, starting from the meaning of “result,” the meaning of “discovery” has also been developed, that is, “new information has been found after a certain process.” This meaning is similar to the basic meaning of “ganqing.” There are 30 such cases, for example:

(31)   听明白了吧, 闹了半天, 是这辆小车违章。

          Ting                                mingbai                 le

          listen                               clear                      (past tense)

          ba,                                   nao le ban tian,

          (exclamation particle)    the truth is

          shi                                   zhe                         liang

          is                                     this                        (measure word)

          xiaoche                           weizhang.

          car                                   break the rules

          *Do you understand now? The truth is that it was this car that broke the rules*.

(32)   闹了半天, 原来是韩女士在更年期的时候没注意补充营养,

          才染上的毛病。

          Nao le bantian,      yuanlai               shi

          the truth is             turn out to be      is

          Han                        nvshi                  zai     gengnianqi

          (family name)        Ms.                    in       menopause

          de                            shihou        mei

          (auxiliary word)      time            not

          zhuyi                       buchong     yingyang,

          pay attention           add             nutrition

          cai        ran        shang       de                           maobing.

          only      incur     get            (auxiliary word)     illness

          *The truth is that Ms. Han didn't pay attention to take in enough nutrition during her menopause, thus developing the illness*.

(33)   隔壁邻居也跟着起急。闹了半天, 罪魁祸首在这儿呢, 看看, 水漏得还挺凶。

          Gebi                        linju          ye

          next door                neighbor    also

          genzhe                    qiji.            Nao le bantian

          together                  worry         the truth is

          zuikuihuoshou

          arch-criminal

          zai                           zher           ne,

          in                             here           (exclamation particle)

          kankan,                   shui            lou

          look                         water         leak

          de

          (auxiliary word)

          hai      ting      xiong.

          still     quite    serious

          *The neighbors next door were also worried together. The truth is, here is the source of the problem. Look, the water is leaking quite seriously*.

Starting from the similar meanings and “sum-up” function, “nao le bantian(guiqi)” has had a development path similar to “hezhe” and “ganqing.” In the corpora, we found 18 cases of “unexpectation” function, which stemmed from the situation that “conclusion” and “discovery” often “violates” the expectations of communication participants, such as:

(34)   记者正纳闷呢, 有人说了, 这其实不是真结婚, 什么？闹了半天是假的？

          Jizhe           zheng                namen

          Reporter     in process of     confused

          ne,                                        youren shuo

          (exclamation particle)          someone say

          le,                   zhe                  qishi            bu      shi

          (past tense)     this                  actually       not     is

          zhen                jiehun,            shenme?

          real                 get married     what

          Nao le bantian     shi     jiade?

          unexpctedly         is       false

          *The reporter was confused, and someone said, this is actually not a real wedding. What? Unexpectedly, this is false?*

(35)   边上有人说了句话, 让我们大感意外。什么？闹了半天白激动啦。

          Bianshang            youren                shuo

          Aside                   someone             say

          le                          ju                        hua,

          (past tense)          (measure word)  words

          rang                     women                da         gan

          let                        we                       very      feel

          yiwai.                  Shenme?

          unexpected          what

          Nao le bantian          bai          jidong      le.

          Unexpectedly           in vain    excited     (past tense)

          *Someone beside me said something that made us feel so surprised. What? Unexpectedly, we had been excited in vain?*

The function of “criticism” which emerged from the “violation” of common sense appeared in 21 cases. For example:

(36)   老百姓说闹了半天, 国家政府、我们选举出来的总统居然骗我们。

          Laobaixing            shuo                    nao le bantian,

          Civilians                say                      ironically

          guojia                    zhengfu,              women

          country                  government         we

          xuanju

          elect

          chulai                    de                         zongtong

          out                        (auxiliary word)   president

          juran                     pian                      women.

          unexpectedly       cheat                      us

          *The civilians said that ironically, the country, the government, and the president we elected unexpectedly cheated us*.

(37)   闹了半天, 原来自己就是井盖的负责人, 自己的井盖子自己都不知道。

          Nao le bantian,                yuanlai                    ziji

          Ironically                         turn out to be          self

          jiushi                               jinggai                     de

          is                                     drain cover              (auxiliary word)

          fuzeren,

          person in charge

          ziji                                  de                             jinggaizi

          self                                 (auxiliary word)      drain cover

          ziji                                  dou                          bu

          self                                 even                         not

          zhidao.

          know

          *Ironically, they themselves are in charge of this drain cover. They even don't know their own drain covers*.

(38)   闹了半天, 全是低层次的、粗鲁的、野蛮的竞争, 你不能提高层次。

          Nao le bantian,        quan                     shi

          Ironically                 all                        are

          di                             cengci                  de,

          low                          level                    (auxiliary word)

          culu                         de,

          rude                        (auxiliary word)

          yeman           de                            jingzheng,

          barbarous      (auxiliary word)     competition

          ni                   buneng                   tigao

          you                can't                       enhance

          cengci.

          level

          *After all, these are all rude and barbarous competitions of a low level, and you can't enhance them*.

There is 6 cases of “humor” due to “violation” of Cooperative Principles:

(39)   敢情就是这么一个法制, 想着法的制人, 这就是他们所谓的法制, 闹了半天, 人家是这么理解的。

          Ganqing                     jiushi                             zheme

          you dare say              is                                    such

          yi                               ge                                   fazhi,

          one                            (measure word)              legality

          xiang                         zhe                                 fa

          think                         (progressive particle)     method

          de                              zhi                                  ren,

          (auxiliary word)        bully                              people

          zhe      jiushi      tamen       suowei

          this      is            they          say

          de                          fazhi,         nao le bantian,

          (auxiliary word)    legality      after all

          renjia     shi      zheme      lijie                 de.

          they       are      so             understand      (auxiliary word)

          *You dare say, this is their ‘legality’, that is, using all methods to bully you. This is the ‘legality they mean. After all, this is their understanding*.

**Figure 5:**
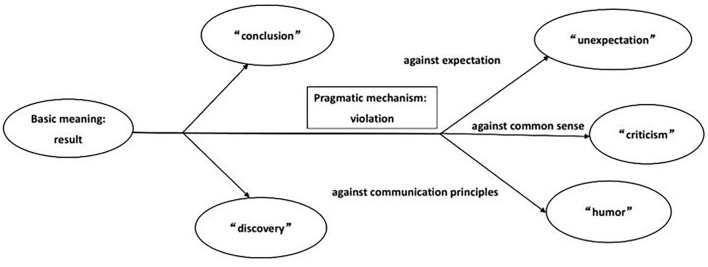
The meanings and pragmatic functions of “nao le bantian(guiqi).”

In (39), “nao le bantian” is also used together with “ganqing,” which proves that they are similar in pragmatic functions and strengthens the effect of ”humor.” To sum up, starting from a simple verb phrase, “nao le bantian (guiqi)” gradually obtains extended meanings similar to the original meanings and basic function of “hezhe” and “ganqing,“ then grammaticalized into a fixed phrase similar to an adverb, and produced pragmatic functions similar to the former two words through verbal communication. The changing path of its semantic meanings and pragmatic functions is shown in Figure 5.

**Figure 6:**
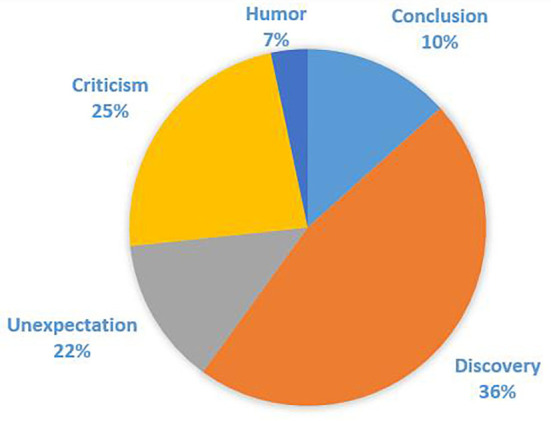
Distribution of “nao le bantian(guiqi).”

The distribution of meanings and pragmatic functions of “nao le bantian(guiqi)” in corpora is shown in Figure 6.

As for “nao le bantian,” the meaning of “discovery” is the most used in the corpora, followed by the function of “criticism” and “unexpectation,” “conclusion” and “humor” still fewer.

### The summary of “sum-up” mood adverbs

So far, we have clarified the semantic and pragmatic functions of the mood adverbs “hezhe” and “ganqing,” as well as the phrase “nao le bantian(guiqi).” We can see that, due to the motivation of verbal communication, words with different original meanings can also accept the influence of the same pragmatic mechanism and “emerge” with similar functions to make them “come to the same goal by different paths.” From the meanings of “conclusion,” “discovery” and “result,” the above three words have moved from “sum-up” function through the mechanism of “violating” some inherent standards, and then produced three corresponding pragmatic functions that not only help to convey information, but also convey a specific tone of the speakers. The distribution of the semantics and pragmatic functions of the three mood words are summarized as in Table 1.

**Table 1:** Distribution of three mood words.

	**Semantics**				**Pragmatic Functions**						**Total**
	**Conclusion**		**Discovery**		**Unexpectation**		**Criticism**		**Humor**		
	**Amount**	**Rate**	**Amount**	**Rate**	**Amount**	**Rate**	**Amount**	**Rate**	**Amount**	**Rate**	
Hezhe	77	21%	–	–	116	31.5%	91	24.7%	84	22.8%	368
Ganqing	–	–	422	50.7%	109	13.1%	147	17.6%	155	18.6%	833
Nao Le Bantian (Nao le Guiqi)	8	9.6%	30	36.1%	18	21.7%	21	25.3%	6	7.3%	83

It should also be noted that the emergence of pragmatic function is a “self-organization” phenomenon spontaneously emerging from communication behavior. It has a certain randomness in which select words are used in peculiar directions and to specific extents. Therefore, not all words with the meaning of “conclusion” will produce the pragmatic function of “violation” (e.g., zongzhi), neither will all words with the meaning of “discovery” evolve along this path (e.g., yuanlai).

To further verify the effect that communication style has on the pragmatic distribution of the above words, we compared corpus 2 with corpus 1. The rate of the total number of characters between corpus 1 (MLC) and corpus 2 (script) was about 330:1, while the ratio of the three adverbs: 1.17:1, 11.1:1, and 4.71:1. It can be seen that these three words appear much more intensively in corpus 2, which is because corpus 2 is created by imitating the dialogue in ordinary family life in Beijing, and therefore such mood adverbs are used more frequently than in interview programs. Compared with the overwhelming advantage of “hezhe” in corpus 1, there is little gap between “hezhe” and “ganqing” in corpus 2, although the former appears a little more, which may be due to the role of the dialect region. “Nao le bantian” is still relatively few, and there is no “nao l guiqi.” The specific distribution of the three words in corpus 2 was compared with the data obtained in corpus 1, as shown in Table 2.

**Table 2:** Distribution of three adverbs in two corpora.

		**Semantic meaning**		**Pragmatic function**		
		**Conclusion %**	**Discovery %**	**Unexpectation %**	**Criticism %**	**Humor**
Hezhe	1	32	–	18	36	14%
	2	25	–	8	42	25%
Ganqing	1	–	49	20	14	17%
	2	–	42	10	37	11%
Nao le bantian (guiqi)	1	14	47	13	23	3%
	2	0	28	44	28	0

It can be seen from Table 2 that in both corpora, the basic meaning of “conclusion” occurs quite often but not in the highest frequency. The most frequently used pragmatic function is “criticism“. However, because corpus 2 is a comedy style, the frequencies of “criticism” and “humor” are higher, and the proportion of “unexpectation” is relatively low. For example:

(40)   这一辆车呀, 也能合着五六万块钱哪。

          Zhe                             yi                                    liang

          this                             one                                  (measure word)

          che                             ya,                                   ye

          car                             (exclamation particle)     also

          neng

          can

          hezhe                         wu                                   liu

          totally                        five                                  six

          wan                            kuai                                 qian

          10,000                       (currency unit)                 money

          na.

          (exclamation particle)

          *This car, totally calculated, is worth 50 to 60 thousand yuan (”conclusion“)*.

(41)   嘿, 合着我这姑妈到现在还不知道有我这么一个人呢。

          Hei,                              hezhe                 wo            zhe

          (exclamation word)     unexpectedly     mine         this

          guma                            dao                    xianzai      hai

          aunt                              till                     now           still

          bu                         zhidao      you     

          not                        know        have

          wo                        zheme       yi         

          I                            such         one

          ge                          ren           ne.

          (measure word)    person      (exclamation particle)

          *Oh my! Unexpectedly, this aunt of mine still doesn't know that I exist (”unexpectation“)*.

(42)   那您呢？ 合着您就什么都不管啦？

          Na       nin           ne?                          Hezhe

          then    you          (question particle)   totally

          nin      jiu           shenme                    dou

          you     just          what                         all

          bu       guan         la?

          no       take on    (question particle)

          *How about you? Totally speaking, you don't take on any affairs at home? (”criticism“)*.

(43)   哎嗨嗨, 合着你们家祖宗是一太监。

          Aiheihei,                      hezhe        nimen         jia

          (exclamation word)     totally        your          family

          zuzong                         shi             yi               taijian

          ancestor                       is               one            eunuch

          *Good heavens, totally speaking, your ancestor was an eunuch (”humor“)*.

”Hehze” in (40) is the conclusion of the money calculation. (41) is the youngest son of the family who is surprised that his aunt doesn't know about his existence. (42) criticizes the attitude of “doing nothing” as a family member, and the obvious impossibility of “the ancestor is an eunuchs” in (43) is to obtain the effect of humor.

Of all “ganqing” in the two corpora, the frequency of the basic meaning “discovery” appears the highest—more than two-fifths. Among the later emerging pragmatic functions, corpus 2 still has more “criticism.” “Unexpectation” and “humor” are slightly lower than those in corpus 1. For example:

(44)   噢我说呢, 敢情这小时候就有事啊。

          O                                     wo                shuo

          (exclamation word)         I                   say

          ne,                                   ganqing        zhe

          (exclamation particle)    the truth is     this

          xiaoshihou     jiu                                  you

          childhood      already                           have

          shi                 a.

          affair             (exclamation particle)

          *Oh, so I see. The truth is, you two had affairs since childhood (”discovery“)*.

(45)   不是你说这么热闹敢情是一倒卧呀？

          Bushi                                 ni               shuo           zheme     renao

          Oh no                                you             say             so           exited

          ganqing                             shi              yi               daowo

          unexpectedly                    is                one             beggar

          ya?

          (exclamation particle)

          *What? You described him so brilliant but unexpectedly he is a beggar? (”unexpectation“)*.

(46)   嘿, 你这不倒霉催的么, 敢情好人全让这局长给干了, 啊, 坏人全让你给当了。

          Hei,                                  ni                   zhe

          (exclamation word)         you                this

          bu                                    daomeicui      de

          not                                   unfortunate    (auxiliary word)

          me,

          (exclamation particle)

          ganqing           haoren          quan        rang         zhe

          you dare say   good man      all           let            this

          juzhang           gei                gan          le,

          director           give              do            (past tense)

          a,                                 huairen       quan       rang

          (exclamation word)    bad guy      all           let

          ni                                 gei             dang       le.

          you                              give            be          (past tense)

          *Hey, you are so unfortunate. You dare say, the Director become the only good man, and just you are the bad guy (”criticism“)*.

(47)   敢情是金子搁哪儿都发光！是葵花长哪儿都向阳！

          Ganqing          shi               jinzi        ge

          You dare say   is                 gold        put

          naer                dou              faguang!

          where             all                shine

          Shi                 kuihua          zhang      naer

          Is                   sunflower     grow        where

          dou                xiang            yang!

          all                  toward         sun

          *You dare say, the gold will shine no matter where you put it! And the sunflower will be toward the sun no matter where you grow it! (”humor“)*.

In (44), “ganqing” indicates the speaker just found some new information. In (45), the speaker was surprised that his family had brought back a beggar. (46) criticized the selfish behavior of the “Director,” and (47) commented on the fact that an elderly man over 60 years old was looking for a lover again. The idiom quoted in (47) was inconsistent with the event itself, so the effect of humor was achieved.

”Nao le bantian” appears fewer times in corpus 2 and has a narrow scope of use. The functions of “conclusion” and “humor” are not used. For example:

(48)   好哇, 闹了半天是你们干的！

          Hao       wa,                                  nao le bantian

          Good     (exclamation particle)    the truth

          shi         nimen                             gan

          is           you                                 do     

          de!

          (auxiliary word)

          *Good! It turned out to be you who did it! (”discovery“)*

(49)   志新:原先我还真以为我喜欢她, 闹了半天……

          Zhixin:   Yuanxian   wo   hai

          (name):   before       I       still   

          zhen        yiwei        wo    xihuan ta,

          really      think         I       like her

          nao le bantian…

          unexpectedly

          胡三:呸！

          Husan: Pei!

          (name): (exclamation word)

          志新:我还真喜欢她……

          Zhixin:     wo   hai    zhen     xihuan   ta…

          (name):     I      still   really   like        her

          *Zhixin: I thought I really liked her before, but it turned out that…*

          *Husan: Oh!*

          *Zhixin: I really like her…(”unexpectation“)*

(50)   我在外头整天奔命似的我给谁奔哪？闹了半天我给你奔哪!我该你的我是欠你的？

          Wo            zai      waitou          zhengtian

          I                in        outside         all day

          benming   shide   wo               gei

          strive        like      I                  give

          shui          ben      na?

          who          strive  (question particle)

          Nao le bantain   wo      gei

          Ironically           I         give

          ni                       ben     na!

          you                    strive  (exclamation particle)

          Wo      gai     nide   wo   shi    qian             nide?

          I          own   your   I      am    in debt         your

          *I strive to earn money all day with my life outside home, and for whom do I do this? Ironically, I strive for you! Do I own you a debt? (”criticism“)*.

Case (48) shows that the speaker has finally discovered the originator of the incident, and the speaker in case (49) is surprised that he suddenly realizes his true feelings. It is worth noting that case (49) inserted another turn between “nao le bantian” and the subsequent unexpected result. This long pause and foreshadowing highlighted the sense of “unexpectation” and achieved dramatic effects. In (50) it is the hostess of the family who criticizes that the housekeeper's salary is too high and thinks that all the money she earns is paid to the housekeeper, which she believes is improper.

To sum up, the previous categorization of the semantic meanings and pragmatic functions of “hezhe,” “ganqing,” and “nao le bantian (guiqi)” has appeared in corpus 1 and 2 in different ways of distribution. Such differences were possibly due to different occasions and genres, and especially due to different origins of dialect regions since corpus 2 depicts the oral communication of people from Beijing, a city that holds its own dialect traits. The above findings evinced the complex nature of oral interaction. Gathering a larger number of cases and differentiating various genres and dialect regions will be an interesting topic for further research.

## Conclusion

This study chose three common adverbs (or phrases) in oral communication, “hezhe,” “ganqing,” and “nao le bantian (nao le guiqi).” Through the analysis of cases from five corpora, it was found that although their original meanings are different, they all go from the basic function of “sum-up” through the mechanism of “violation” in verbal communication and produce the pragmatic functions of “unexpectation,” “criticism,” and “humor.” We categorized such words as “sum-up“ mood adverbs.

Ancient and contemporary corpus statistics reveal the origins and distributions of different functions of the three words, and the comparison between MLC and script corpus also shows that the distribution proportion of various functions will be different on the premise of different communication nature and purpose. For example, the proportion of “critical” and “humor” functions tends to rise in comedy scripts with satirical and humorous effects.

The conclusion of this paper reflects the influence of verbal communication on the meaning and function of words. Through the analogy of similar psychological mechanisms, words with different original meanings may “emerge” with similar pragmatic functions through long-term use. Especially for the category of mood words, which is inseparable from the communication process and highlights the vividness of oral language, it is necessary to break the traditional simple classification based on semantics and explore their function and usage from practical communication. In the future, it is expected to expand the research scope to more types of oral corpora to reveal the influence of communication modes on the distribution of word functions, considering the factor of the genre, dialect, etc. And there are still other words and phrases that could obtain a similar “sum-up” function and mood functions to the words considered in the paper, such as “shuo dao di” (说到底, to speak to the bottom), and “shuo bai le” (说白了, to speak clearly), the study of which could enrich the category of “sum-up” and become the resources of comparison among such words.

In addition, in TCSOL, more attention should be paid to words from dialects such as “hezhe,” “ganqing” and “nao le bantian (nao le guiqi).” At present, only few studies such as Ding (2003) have discussed the teaching of dialect words. In second language learners' Chinese discourse, as in HSK corpus, neither “hezhe” nor “ganqing” as adverbs has been used, while “nao le bantian” appeared only once, in original meaning as a verbal phrase:

(51)   可是闹了半天, 究竟敌不过爸爸, 她只好哭一阵。

          keshi    **nao le bantian**,                         jiujing      di

          but       make a fuss for quite a while    at last       contend

          bu                guo     baba,

          not               over    father

          ta                 zhihao    ku      yi zhen

          she              only        cry     a while

          *She made a fuss for quite a while but at last couldn't defeat my father, and she could only cry for a while*.

With the development of China's international exchange, the continuous expansion of high-level Chinese learners, and the increasing use of film, television, and entertainment works as materials for Chinese teaching, more and more learners will be widely exposed to daily used oral words. Some of these words have entered the vocabulary syllabus, and more are to be included. Therefore, based on the conclusions of this research, dialect words can be appropriately selected and supplemented in TCSOL classes, especially for speaking classes of intermediate and advanced levels, and the instruction design of such words can be carried out in combination with their pragmatic functions to reflect the practical and communicative principles of TCSOL.

## Data availability statement

The original contributions presented in the study are included in the article/supplementary material, further inquiries can be directed to the corresponding author.

## Author contributions

The author confirms being the sole contributor of this work and has approved it for publication.

## Conflict of interest

The author declares that the research was conducted in the absence of any commercial or financial relationships that could be construed as a potential conflict of interest.

## Publisher's note

All claims expressed in this article are solely those of the authors and do not necessarily represent those of their affiliated organizations, or those of the publisher, the editors and the reviewers. Any product that may be evaluated in this article, or claim that may be made by its manufacturer, is not guaranteed or endorsed by the publisher.

## Footnotes

^1^It should be noted that “nao le bantian” is actually a phrase in the perspective of traditional grammar. Yet in this study it perform the pragmatic functions as an integrated entity, just like so-called “discourse marker.” So for the convenience of narrating, in this paper we will call it “mood adverb” as well.
